# Artificial Intelligence and the Transformation of Cell and Gene Therapy Development

**DOI:** 10.3390/pharmaceutics18030356

**Published:** 2026-03-13

**Authors:** Jared R. Auclair, Jeewon Joung, Maya A. Singh, Gaël Debauve, Rominder Singh

**Affiliations:** 1 College of Professional Studies, Northeastern University, Boston, MA 02115, USA; 2 National Institute of Food and Drug Safety Evaluation, Ministry of Food and Drug Safety, Cheongju-si 28159, Republic of Korea; jeewon@korea.kr; 3 Department of Bioengineering, University of Washington, Seattle, WA 98195, USA; 4 Gene Therapy Analytical Sciences, UCB, 1070 Brussels, Belgium

**Keywords:** artificial intelligence, machine learning, cell and gene therapy, CGT development, CGT manufacturing

## Abstract

Cell and Gene Therapy (CGT) represents a paradigm shift in medicine, offering curative potential for previously intractable diseases. However, the complexity, high cost, and manufacturing challenges inherent in developing, producing, and administering these therapies hinder their widespread accessibility. This review examines the critical and increasingly synergistic role of Artificial Intelligence (AI) and Machine Learning (ML) in overcoming these barriers across the entire CGT lifecycle, from discovery and construct design to smart manufacturing, clinical translation, and regulatory applications. We analyze how AI-driven approaches fundamentally differ from conventional methods, facilitating rapid construct optimization, generating highly predictive translational models, enabling the vision of autonomous, digital-twin-driven manufacturing, and establishing new paradigms for pharmacovigilance and regulatory oversight. The integration of AI is not merely an incremental improvement but a foundational transformation, positioning CGT to move from niche, bespoke treatments to scalable, accessible, and highly personalized medical modalities. We conclude by discussing current gaps, particularly data scarcity and regulatory uncertainty, and outlining a roadmap to realize the full potential of AI-enabled CGT.

## 1. Introduction

Cell and gene therapies encompass a spectrum of products, from living cell infusions without genetic modification to gene-edited immune cells and viral-vector-based DNA therapies. This diversity creates both shared and distinct translational and manufacturing challenges [1,2]. The Cell and Gene Therapy (CGT) sector is experiencing unprecedented growth [3,4]. As of late 2025, there were over 3200 active clinical trials investigating gene, cell, or RNA therapies globally, indicating a robust and expanding pipeline at various stages of clinical development worldwide [5]. This therapeutic domain is expected to grow dramatically, with the global gene therapy market projected to expand from an estimated USD 8–11 billion in 2025 to USD 49–118 billion by 2035 (a compound annual growth rate (CAGR) of 19–24% across that decade) [6]. Furthermore, market analyses project cell therapy to become the third-largest modality in oncology by 2030, trailing only small molecules and antibodies, underscoring its immense clinical and commercial potential [7]. This growth is driven by unprecedented clinical success in areas such as hematological malignancies and the potential to treat monogenic disorders [8].

### 1.1. What Is Cell and Gene Therapy

Cell and gene therapies encompass a diverse and revolutionary range of therapeutic modalities that aim to restore, modify, or replace biological function by targeting the cell or correcting genetic defects at the molecular level. These therapies are often categorized into three overlapping domains: cell therapies, gene therapies, and combined cell-gene approaches.

Cell therapies involve the administration of live cells—either autologous or allogeneic—that are minimally or more than minimally manipulated, such as cultured mesenchymal stem cells (MSCs) or natural killer (NK) cells, which is discussed in detail as part of the US FDA guidelines [9]. A key example of cell therapy is CAR-T, in which the T cells are genetically engineered to express a chimeric antigen receptor (CAR) designed to recognize a specific antigenic epitope expressed on target cells. Importantly, these target antigens are not always uniquely restricted to malignant cells. For example, anti-CD19 CAR-T cells are used to treat B-cell malignancies by targeting CD19, a surface antigen expressed on malignant B cells, but they also eliminate normal CD19-positive B cells. Gene therapies, in contrast, involve introducing, modifying, or removing genetic material within a patient’s cells to treat or prevent disease by altering gene expression or correcting faulty genes.

Gene therapy can be delivered in two ways:

- In vivo gene therapy: The genetic material is introduced directly into the patient’s body, typically using viral vectors such as adeno-associated virus (AAV). This approach targets cells within the body without prior extraction [10].
- Ex vivo gene therapy: Cells are harvested from the patient (or a donor), genetically modified outside the body, often using lentiviral or retroviral vectors, and then reinfused. In ex vivo gene therapy strategies, the therapeutic effect comes from the genetic correction or addition, not from the cell type itself (gene-centric) [11].

Cell-gene therapies bridge these two modalities with a clear purpose to give cells a new function (cell-centric), not just correct a gene [12]. Examples include Chimeric Antigen Receptor T-cell (CAR-T, where T cells are engineered to express chimeric antigen receptors) and T-Cell Receptor (TCR-T, engineered to recognize specific antigens) therapies, which involve harvesting a patient’s cells, genetically modifying them ex vivo, and reinfusing them to achieve targeted therapeutic effects [13].

The scientific, operational, and regulatory demands of these approaches vary significantly depending on the source of cells, the type of genetic modification (if any), and the intended mechanism of action. Tailored strategies are required across the translational and manufacturing pipeline to accommodate this diversity, from potency assays and stability requirements to vector design and cell viability parameters.

It should be noted that cell and gene therapy differ from traditional biologics and small molecule drug development across multiple dimensions, from discovery and translational research to clinical development, manufacturing, logistics and regulatory oversight. These differences highlight the profound challenges CGT faces, which are difficult to address using conventional pharmaceutical methods. Details are provided in Table 1.

### 1.2. Translational Differences Between Cell Therapy & Gene Therapy

Cell and gene therapies, while often grouped together, differ significantly in their biological underpinnings and translational considerations. Cell therapies such as mesenchymal stem cells (MSCs) or induced pluripotent stem cells (iPSCs) typically rely on their original cell functions. In contrast, gene therapies, whether ex vivo or in vivo, involve precise genetic interventions that introduce or edit genes using viral or non-viral vectors. The key determinants of potency differ between cell therapies and gene therapy: in cell therapies, therapeutic effect hinges on cell viability, phenotype, and immunomodulatory function, whereas in gene therapy, efficacy depends on vector transduction (infectivity), transgene expression levels and delivery efficiency [14].

A deep dive into gene therapy reveals additional complexities. For in vivo approaches, vector biodistribution, transduction efficiency, and durability of transgene expression are critical determinants of efficacy and safety. Analytical assays must therefore include sensitive methods for vector genome quantification and long-term monitoring of transgene expression in target and non-target tissues. Furthermore, the risk of insertional mutagenesis, particularly with integrating vectors, necessitates advanced genomic analyses to detect rare but potentially oncogenic events. Immunogenicity is also a central concern, as pre-existing or therapy-induced immune responses against vector capsids or the transgene product can limit efficacy and pose safety risks.

These challenges, along with the broader differences between cell and gene therapy, shape the analytical and regulatory focus as summarized in Table 2. Cell therapy assays prioritize identity, purity, and functional immunomodulation, while gene therapy assays center on vector copy number, product purity (including % full capsid and process-related impurities), off-target editing risks, and long-term expression durability. Regulatory agencies accordingly scrutinize distinct aspects of each modality, especially cell therapies for expansion protocols and safety, and gene therapies for vector design, insertional mutagenesis, and immune activation. Together, these distinctions underscore the need for tailored development and regulatory strategies for each platform.

### 1.3. The Inherent Complexity of CGT

Despite its promise, the CGT development pipeline is fraught with challenges that conventional pharmaceutical development methods are ill-equipped to handle:

- Biological Complexity: CGTs involve living matter. The behavior, potency, and safety profile of a modified cell or gene construct are highly dependent on complex, non-linear biological interactions that are difficult to predict a priori.
- Manufacturing Intricacy (The Vein-to-Vein Challenge): Especially for autologous cell therapies like CAR-T, manufacturing is a highly complex, patient-specific process. This complex, high-variability process is costly, time-consuming, and highly susceptible to batch failures, which could result in critical delays for patients. When failures occur, starting material often must be re-collected, which adds another risk for patients who are already immunocompromised and may not be clinically stable enough for another harvest [15,16].
- Scale-Out Complexities: CGT manufacturing often relies on scale-out rather than traditional scale-up, requiring the simultaneous operation of many small, parallel production runs. This introduces substantial challenges in maintaining consistency, facility throughput, staffing, scheduling, and quality control across hundreds to thousands of individualized batches, each effectively its own “mini-manufacturing run.”
- Translational Risk: Moving from in vitro or animal models to human clinical trials often reveals unexpected toxicity or efficacy issues due to the vast differences between model systems and the human physiological environment.
- Regulatory Uncertainty: The novelty and complexity of CGTs necessitate new frameworks for regulatory oversight, including product classification and donor eligibility. Particularly when integrating cutting-edge manufacturing controls and AI-driven quality systems.

### 1.4. How AI Contributes to CGT Discovery, Development, Manufacturing, and Lifecycle Management

Artificial intelligence, encompassing Machine Learning (ML), Deep Learning (DL), and advanced computational techniques, offers a critical solution to these complexities. AI excels at identifying non-obvious patterns, predicting outcomes in high-dimensional datasets, and optimizing multi-variable systems. These capabilities are nicely matched to the challenges of CGT. Importantly, the value of AI extends beyond discrete applications in design or manufacturing; it has the potential to support end-to-end lifecycle management. From early construct optimization and translational modeling to adaptive manufacturing control, real-time quality monitoring, post-approval surveillance, and long-term outcome prediction, AI can integrate data streams across traditionally siloed domains. In this way, AI enables a continuous learning framework in which insights from clinical performance inform manufacturing refinement, safety monitoring guides process controls, and real-world evidence contributes to iterative product optimization. The interest in the intersection of AI and CGT stems from the recognition that only advanced computation can unlock the speed, precision, and scalability required to transform these therapies from bespoke interventions into accessible, mainstream therapies [17].

### 1.5. AI-Enabled CGT Development vs. Conventional Approaches

The shift to an AI-driven paradigm can be transformative, replacing slow, iterative, trial-and-error processes with predictive, data-driven workflows. This difference is noticeable across various stages of the development and delivery of these therapies (Table 3).

Table 3 illustrates a fundamental shift from conventional, trial-and-error–based CGT development toward a predictive, AI-enabled framework that spans the entire therapeutic lifecycle. In research and design, AI narrows candidate selection through predictive modeling and generative tools rather than relying on large experimental libraries. During translational development and manufacturing, digital twins and real-time analytics replace reactive monitoring with proactive drift detection and adaptive process control. In clinical development and pharmacovigilance, AI supports enriched patient selection, adverse event prediction, and automated signal detection across integrated data streams. Finally, regulatory engagement evolves from static, submission-based review toward context-of-use validation and continuous performance monitoring, reflecting a more dynamic and data-driven oversight model.

## 2. AI in R&D for CGT: Design and Optimization of Constructs

The foundation of any successful CGT lies in its therapeutic construct, the design of the viral vector, the genetic payload, or the Chimeric Antigen Receptor (CAR). Conventional construct design is highly laborious, relying on synthesizing and testing large libraries of variants. AI, particularly generative models and active learning, introduces an in silico paradigm that drastically accelerates this process [18]. In addition to the expanding role of gene therapy, cell therapy has emerged as a transformative pillar in regenerative medicine, offering curative potential for conditions previously deemed intractable. Autologous cell therapies, such as CAR-T, present unique translational challenges due to their inherently personalized nature. Each patient’s cells must be harvested, engineered, and reinfused under tightly controlled conditions, requiring a complex “vein-to-vein” supply chain to ensure a chain of identity and product integrity. Meanwhile, allogeneic cell therapies promise scalability but face variability in donor profiles and require robust immunologic controls.

### 2.1. Generative Design for Novel Constructs

AI is transitioning construct design from an evolutionary process (testing modifications of existing designs) to a generative one (creating entirely novel, optimized designs).

- Vector Optimization: Viral vectors, such as Adeno-Associated Virus or lentiviruses, are crucial for gene therapy delivery [19]. AI models can predict the tropism (which tissues or cells the vector targets) and immunogenicity (the likelihood of an adverse immune response) based on the vector’s capsid protein sequence. Machine learning approaches also optimize promoter selection, codon usage, and regulatory elements to maximize transgene expression while minimizing off-target effects. Generative AI can design novel capsid variants with superior tissue specificity and reduced immunogenicity, circumventing the need for blind high-throughput screening [20]. These combined strategies enable rational design of next-generation vectors with improved safety and efficacy, particularly for in vivo gene therapies where precise targeting and long-term expression are essential.
- CAR Structure Design: For CAR-T cells, the sequence of the CAR dictates its binding affinity to the target, its signal transduction strength, and ultimately, the cell’s persistence and killing efficacy. Machine learning models, utilizing large datasets of receptor sequences and their corresponding in vitro functional outcomes, can predict the optimal amino acid sequences for the single-chain variable fragment (scFv), hinge, and co-stimulatory domains. This includes predicting the necessary balance between high binding affinity and low tonic signaling, a critical factor for CAR-T safety and efficacy. Recent work shows that deep-learning frameworks can design peptide binders directly from sequence by using language-model embeddings and contrastive learning to identify high-affinity candidates. This capability streamlines and accelerates the generation of targeted peptide components that may enhance next-generation cell therapy applications [21,22,23].
- AI is emerging as a powerful tool in the design and optimization of nucleotide delivery systems, particularly lipid nanoparticles (LNPs) and polymer-based vectors used for mRNA and gene therapies. Unlike traditional formulation approaches that rely on factorial design-of-experiments, AI models can capture nonlinear relationships among lipid composition, particle morphology, encapsulation efficiency, biodistribution, and toxicity [24]. Machine learning and generative chemistry frameworks have been applied to design novel ionizable lipids with optimized pKa values and biodegradability profiles, while Bayesian optimization and active learning strategies reduce the experimental burden required to identify high-performing formulations [25]. Importantly, AI enables integration of sequence-level features such as mRNA length, GC content, and secondary structure with formulation variables, allowing predictive modeling of stability, endosomal escape, and tissue targeting [26]. These approaches move delivery science from empirical optimization toward a mechanistically informed, data-driven paradigm, with potential to improve therapeutic index, manufacturability, and scalability of nucleotide-based CGTs.

### 2.2. The Computational-Experimental Closed Loop (DBTL)

The most advanced applications utilize a Design-Build-Test-Learn (DBTL) loop, where computational modeling is integrated directly into the synthetic biology workflow [9].

- Design (In silico): AI tools perform in silico mutations or sequence generation at the DNA, mRNA, and protein levels.
- Prediction (In silico Structure/Function): AI models predict properties of the newly designed sequences:
  - DNA: Presence of regulatory elements, off-target gene editing effects (for CRISPR applications) make DNA design in CGT a highly repetitive, labor-intensive process. Integrating AI tools to design primers, optimize DNA sequences, and balance GC content can significantly streamline this otherwise manual and time-consuming workflow.
  - mRNA: GC content, minimum free energy (related to stability). AI solutions select precise guide RNA (gRNA) sequences, reducing off-target effects and making gene editing safer.
- Protein: Stability, binding affinity (e.g., to the target antigen), and experimental endpoints like efficacy or toxicity. Learn (Active Learning): The resulting experimental data (e.g., transcriptomics, protein presence, toxicity scores) is fed back into the AI model. This Active Learning cycle refines the model’s predictive power, making the next generation of in silico designs even more efficient and accurate. This integrated process drastically shrinks the multi-year timeline associated with conventional construct optimization

**Figure 1:**
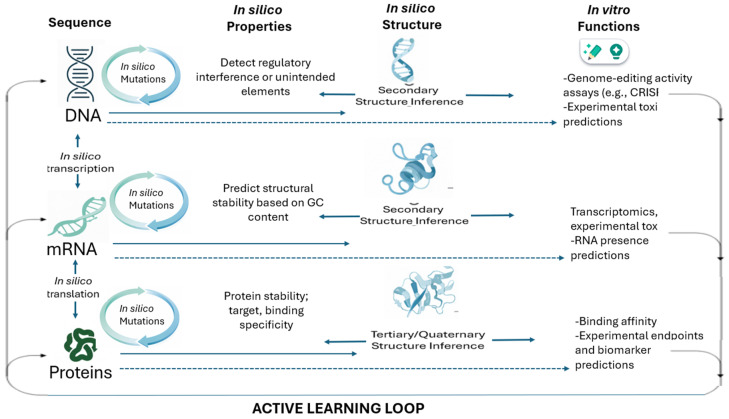
Machine learning-driven closed-loop, sequential design for cell and gene therapy.

Figure 1 illustrates a continuous, AI-enabled learning loop that replaces traditional linear, trial-and-error therapeutic design with an iterative, data-driven process. Rooted in the central dogma of molecular biology, computational models simulate how modifications at the DNA level propagate to mRNA and protein, enabling large-scale in silico mutation, structural prediction, and functional forecasting before laboratory experimentation. AI systems evaluate properties such as regulatory elements, RNA stability, protein folding, binding affinity, and potential toxicity, effectively conducting rapid virtual prototyping across molecular layers. Experimental in vitro results, including genome-editing efficiency, transcriptomic profiles, and protein functionality, are then fed back into the models, refining their predictive accuracy through active learning. This closed-loop architecture accelerates discovery, reduces experimental burden, and enhances safety by identifying suboptimal or high-risk candidates early in development, representing a more efficient and adaptive paradigm for CGT research.

## 3. CGT Translational Studies and Modeling: Predicting Human Outcomes

A major hurdle in CGT development is the limited predictability of preclinical models, such as animal studies, for human safety and efficacy. AI addresses this by building sophisticated digital twins and in silico models that simulate human physiology and patient-specific responses.

### 3.1. Digital Twins for Therapeutic Simulation

Digital twins, in the context of CGT, are virtual representations of a patient’s biological systems [27], created by integrating diverse, multimodal data:

- Multimodal Data Integration: These models integrate omics data (genomics, transcriptomics, proteomics, metabolomics), imaging data (radiology, pathology), and clinical data (EHRs, patient history). Machine learning algorithms process this high-dimensional, heterogeneous data to create a unified, predictive profile of how an individual patient might respond to a specific CGT product.
- Simulating Efficacy and Safety: By modeling the immune system’s interaction with the therapy, AI can simulate critical outcomes:
  - Efficacy: Predicting the persistence and proliferation of CAR-T cells in vivo, and their tumor-killing kinetics.
  - Safety: Predicting the risk of serious adverse events like Cytokine Release Syndrome (CRS) and Immune Effector Cell-Associated Neurotoxicity Syndrome (ICANS). AI solutions predict individual patient risk from multi-omics data, allowing for proactive management and improved outcomes. This is exemplified by frameworks like CART-GPT, an AI linguistic framework that uses T-cell information to interpret neurotoxicity and therapeutic outcomes [28].
- Advanced Simulation for Gene Therapy: In gene therapy, digital twins can be leveraged to simulate vector biodistribution, transgene kinetics, and immune responses in silico, based on patient-specific anatomical and molecular data [29]. This enables the prediction of therapeutic windows, identification of patients at risk for adverse events, and optimization of dosing regimens. AI-powered models can also integrate longitudinal biomarker data to monitor transgene persistence and detect early signs of vector-related toxicity, supporting proactive clinical management.

### 3.2. Predictive Biomarkers and Patient Selection

AI-driven models are indispensable for precision in patient selection, particularly given the high cost and potential toxicity of CGTs.

- Pre-treatment Risk Prediction: ML models can analyze patient omics data (e.g., baseline inflammatory markers, cell repertoire features) to assign a toxicity risk score before the patient undergoes conditioning therapy and infusion [30]. This allows clinicians to tailor conditioning regimens, select appropriate bridging therapies, or even pre-emptively administer mitigating drugs, thereby improving safety and personalized patient management.
- Enhancing Clinical Trial Design: AI-enriched patient selection identifies subgroups most likely to respond positively, maximizing the statistical power and efficiency of small population CGT trials. Furthermore, AI enables adaptive trial designs, where the protocol is dynamically adjusted based on accumulating data. The use of virtual or external controls, derived from historical patient data analyzed by AI, can also reduce the need for large, traditional control arms, accelerating development [31].

### 3.3. AI and Reimbursement Modeling in Gene and Cell Therapy

Gene and cell therapies frequently command *one-time* or *lifetime managed-care payments* that are orders of magnitude higher than traditional small molecules or biologics, necessitating innovative reimbursement strategies. Traditional pricing and reimbursement frameworks struggle to accommodate outcomes that may unfold over years or decades, and they must reconcile high upfront costs with long-term benefit. In response, stakeholders are beginning to explore how advanced computational tools, including AI and ML, can inform more sophisticated, data-driven reimbursement models.

AI is being applied to develop predictive economic models that estimate long-term value, cost savings, and risk-sharing potential for CGTs. These models leverage real-world evidence, longitudinal health outcomes data, claims histories, and multimodal clinical datasets to project lifecycle costs and patient benefits. The ability of AI to integrate heterogeneous data streams and detect complex patterns enables the generation of more accurate forecasts of therapy durability, quality-adjusted life years (QALYs), and healthcare resource utilization, which are key inputs for value-based payment arrangements [32].

For example, industry forecasts such as these have highlighted AI as an emerging enabler for economic modeling and payer engagement [33,34]. These reports identify AI-driven actuarial and simulation frameworks that help payers evaluate alternative payment pathways, such as annuity payments, outcomes-based contracts, or “pay-for-performance” arrangements, in which reimbursement is tied to post-therapy clinical outcomes. AI models also assist in identifying subpopulations most likely to benefit from a given therapy, thereby supporting risk stratification that underpins differential pricing or conditional reimbursement.

Importantly, this use of AI is not limited to retrospective cost analysis; it supports prospective scenario planning that informs public policy and payer negotiations before therapies are launched. By projecting the financial impact of different reimbursement models under varying assumptions of durability, durability of response, and long-term toxicity risks, AI tools enable more nuanced policy discussions between manufacturers, payers, and regulatory bodies [35].

Incorporating this dimension into CGT development recognizes that economic and access outcomes are part of the broader therapeutic lifecycle. As the volume of long-term follow-up and real-world evidence grows, AI-powered reimbursement modeling will become increasingly central to ensuring that breakthrough CGTs achieve sustainable patient access without compromising fiscal solvency.

## 4. Smart Manufacturing with Digital Twins and Machine Learning

The manufacturing of CGTs, particularly the bespoke nature of autologous therapies, presents the biggest hurdle to scalability and cost reduction. The shift to a Smart Manufacturing paradigm, driven by Digital Twins and Machine Learning, is the industry’s critical response to this challenge [36,37]. AI technologies offer novel solutions to address these translational bottlenecks. For instance, digital twins, virtual replicas of manufacturing processes or even individual patients, can model cellular expansion kinetics and simulate product behavior under varying conditions. This allows developers to optimize yield and consistency before actual production begins. Predictive machine learning models can also be trained on manufacturing data to identify critical process parameters and anticipate batch failures, significantly reducing downtime and improving quality assurance.

### 4.1. The Concept of the Digital Twin in Biomanufacturing

A Digital Twin in biomanufacturing is a real-time, virtual replica of the physical manufacturing process, such as a bioreactor run. This digital model is fed continuous data from biosensors, at-line analyzers, and process control systems, providing a flexible framework for Advanced Therapy Medicinal Product (ATMP) production [27].

- Process Simulation & Optimization: Before a physical manufacturing run begins, an AI-driven digital twin can simulate thousands of “what-if” scenarios. Developers can virtually test variations in raw material sources, temperature profiles, or nutrient feeding strategies, identifying the optimal process parameters for maximizing yield and Critical Quality Attributes in silico. This simulation capability significantly de-risks the process development phase [38].
- Real-Time Monitoring and in-line Quality Control: AI systems integrate with advanced biosensors to monitor Critical Process Parameters like glucose, CO_2_/O_2_ levels, and cell viability in real time.
  - Predictive Quality Control: The digital twin continuously analyzes real-time sensor data to predict key quality attributes (e.g., cell viability, product potency) before the process is complete. AI algorithms detect subtle anomalies or drift trends that might lead to a failed batch, allowing for proactive intervention before a batch is lost.
  - Dynamic Process Control: If the AI detects a process deviation, it can autonomously send commands back to the manufacturing equipment (e.g., adjusting nutrient flow rates) to maintain optimal conditions. This enables the transition to highly efficient and standardized autonomous manufacturing, utilizing tools like reinforcement learning for optimization [6,22,37,39,40,41].
- Integration of AI for Data Harmonization and Advanced Analytics:
  - AI plays a critical role in optimizing the use of process and analytical data by making connections that are currently challenging to establish. A fundamental issue in biomanufacturing is that data are generated from multiple software systems with non-harmonized structures, making trending, analysis, and meta- or multivariate analyses difficult. AI can harmonize data presentation across disparate sources while maintaining data integrity—a prerequisite under GMP principles—ensuring that data are not modified during this process.
  - Harmonized data across software systems can also enable automated report generation (e.g., for method development or product stability) and real-time evaluation of method performance. Instead of relying on a static snapshot from traditional validation at a single point in time, AI could continuously monitor and analyze method performance over time, automatically generating insights and alerts.
  - In the near term, AI could support lower-value-added functions such as automated data double-checking and verification, improving data quality and compliance. Looking forward, AI has the potential to enable automated and unbiased data processing and interpretation, supporting advanced meta-analytical capabilities. However, this requires training AI models with appropriate and representative datasets—a significant challenge in cell and gene therapy due to limited prior knowledge, lack of platform products, small batch numbers, and few products currently on the market.

### 4.2. Optimizing the CAR-T Process & Machine Learning

The CAR-T manufacturing process is highly sensitive to variability. From growing the vector cells to the T cell isolation, machine learning models are applied to individual steps to ensure consistency:

- Cell Expansion Prediction: ML models trained on historical batch data (including initial cell quality, donor characteristics, and media components) can accurately predict the final cell yield and optimal harvest timing. This reduces the risk of process failure and improves product consistency.
- Predictive Maintenance: ML analyzes sensor data from the manufacturing equipment itself to forecast when components require maintenance. This proactive approach prevents costly, unplanned downtime and production interruptions.

### 4.3. End-to-End Supply Chain and Traceability

For autologous therapies, the supply chain is synonymous with patient safety. Digital twins track the entire “vein-to-vein” journey. AI monitors the product’s location, temperature, and integrity from collection to infusion, predicting and preventing deviations that could compromise the final therapy [42,43]. AI is also vital for managing complex, decentralized production facilities through multi-agent scheduling and forecasting critical inputs, such as reagent supply [16,37]. This end-to-end digitization is essential for ensuring product integrity, maintaining the Chain of Identity, and building public confidence.

## 5. Regulatory Frameworks for AI in CGT

The rapid adoption of AI in CGT, particularly in high-risk areas like manufacturing quality control and personalized adverse event (AE) prediction, necessitates the evolution of regulatory frameworks. Regulators are adapting to ensure that AI-driven processes meet the equivalent safety, quality, and efficacy standards as conventional methods, while also facilitating innovation [44,45].

Gene therapy products, particularly those using novel vectors or genome editing technologies, often face additional regulatory scrutiny regarding long-term safety, vector integration, and germline transmission risks. Regulatory agencies are increasingly requiring comprehensive preclinical studies, long-term follow-up protocols, and advanced analytical methods to assess vector biodistribution, integration sites, and off-target effects. AI tools that enhance the sensitivity and specificity of these analyses are likely to play a growing role in regulatory submissions and post-marketing surveillance for gene therapies.

### 5.1. Evolving Regulations and Key Initiatives for CGT

The U.S. Food and Drug Administration (FDA) is actively engaging with the intersection of AI and manufacturing:

- Framework for Regulatory Advanced Manufacturing Evaluation (FRAME) Initiative: The FDA’s FRAME program is crucial for assessing new manufacturing technologies, including those powered by AI. This initiative seeks to facilitate the adoption of advanced manufacturing techniques to improve drug quality and supply chain resilience [40].
- Cross-Center Strategy (2024): The FDA’s medical product centers outlined a unified strategy for AI across the product lifecycle. Center for Biologics Evaluation & Research (CBER), which regulates biologics like CGTs, has issued preliminary guidelines emphasizing risk management for AI applications in biologics production [40].
- AI-Derived Data for Regulatory Review: The FDA has issued guidance on “Considerations for the Use of Artificial Intelligence to Support Regulatory Decision-Making for Drug and Biological Products” [46]. This focuses on the standards for submitting and reviewing AI-derived data, ensuring that the results and predictions generated by these models are trustworthy, traceable, and understandable to reviewers.

European regulatory bodies, notably the European Medicines Agency (EMA), are similarly adapting the regulatory pathway for Advanced Therapy Medicinal Products in the EU, which is distinct and continues to evolve to accommodate technological advancements. Studies indicate that regulatory measures and guidance are continuously refined to streamline approval timelines for ATMPs [21,44].

The World Health Organization (WHO) provides foundational guidance for the regulation of cell and gene therapies (CGTs). Its document *Considerations on Regulatory Convergence of Cell and Gene Therapy Products* (WHO/CGTPs/Draft, December 2021) outlines definitions, risk-based regulatory principles, and recommended standards for quality, safety, and efficacy. WHO’s “Cell, Tissue and Gene Therapy Products, Standards and Specifications” serves as a public framework to help harmonize regulatory expectations globally. Citing these WHO documents in CGT development underscores the importance of regulatory convergence and encourages alignment with international best practices.

### 5.2. Regulating the Evolving Regulatory Landscape

Regulators and scholars have begun to argue that AI in therapeutics should be viewed at the ecosystem level rather than as isolated tools. The concept of an AI-Enabled Ecosystem for Therapeutics (AI2ET) describes the full set of AI-enabled systems, platforms, processes, and products that underlie modern drug development, providing a necessary framework for comprehensive regulatory oversight [46]. Moreover, in the absence of a well-defined regulatory framework for an AI-enabled ecosystem in human therapeutics, a risk-based approach should be adopted to regulate this evolving field.

Regulatory oversight of artificial intelligence in CGT is evolving within existing drug, biologics, and medical device frameworks rather than through CGT-specific AI regulations. In the United States, the FDA’s AI/ML-Based Software as a Medical Device (SaMD) framework and its 2025 draft guidance on AI-enabled device software functions emphasize lifecycle management, risk-based validation, and post-market performance monitoring [47]. While primarily device-focused, these principles are increasingly relevant to AI systems used in CGT manufacturing analytics and clinical decision support [48].

More directly applicable to CGT sponsors is the FDA’s 2025 draft guidance on the use of AI to support regulatory decision-making for drugs and biological products [49], which introduces a credibility framework tied to the model’s defined “context of use.” This is particularly relevant for AI-driven comparability assessments, predictive release testing, digital twin–enabled process optimization, and toxicity modeling. Similarly, the EMA’s 2024 reflection paper on AI in the medicinal product lifecycle underscores data governance, transparency, validation, and early regulatory engagement when AI materially influences development or manufacturing decisions [50].

## 6. AI-Enabled Pharmacovigilance and Safety Monitoring

Post-market surveillance (pharmacovigilance) for CGTs is uniquely challenging. Adverse events can be severe (e.g., secondary malignancies, delayed neurotoxicity) and can be sudden or occur years after infusion. Traditional pharmacovigilance relies on manual processes, which struggle to detect novel or subtle safety signals.

### NLP and ML for Signal Detection

AI significantly enhances pharmacovigilance by expanding the scope of data analyzed and accelerating signal detection [51]. For example,

- NLP-Driven Case Processing: Natural Language Processing (NLP) can automatically process adverse event reports, patient narratives, and unstructured data from Electronic Health Records (EHRs) and registries. This capability drastically reduces the manual labor required for case intake and preparation.
- ML-Based Signal Detection: Machine Learning algorithms can detect safety signals across multiple, disparate data streams, including EHRs, patient registries, manufacturing data, and patient-reported outcomes. ML models are superior to traditional statistical methods for detecting subtle or complex patterns of AEs that may only manifest in specific patient subgroups or correlate with a manufacturing deviation.
- Linking Manufacturing to Outcome: One of the most powerful applications is linking detailed manufacturing process data (from the digital twin) to long-term patient safety and efficacy outcomes. AI can determine if a deviation in a bioreactor run five years ago is statistically correlated with a late-onset AE in a subset of patients, creating a closed-loop quality system that extends far beyond the point of product release.

## 7. Gaps, Challenges, and the Future Outlook

While the promise of AI in CGT is immense, its full realization is contingent on overcoming several significant operational, technological, and regulatory hurdles.

### 7.1. Gaps and Challenges

- Data Scarcity and Quality: This is the most fundamental bottleneck. CGT data is inherently scarce (due to small patient populations) and often remains siloed across institutions. The quality is inconsistent, lacking standardization in collection, annotation, and storage. Robust AI models require vast amounts of high-quality, structured data to train, hindering the generalizability and reliability of many current ML applications. Moreover, due to the small clinical data of GCT, RWD/RWE is widely used for data validation. However, the regulation of personal data protection (i.e., the level of protection) is different from country to country; it is sometimes difficult to collect sufficiently qualified data. This limits the data utilization and validation. For gene therapy, these challenges are even more pronounced: the rarity of target diseases and the need for long-term follow-up exacerbate scarcity, while heterogeneity in vector platforms, patient populations, and clinical endpoints complicates data aggregation and model generalizability. To unlock the full potential of AI-driven analytics, collaborative data-sharing initiatives and the development of a standardized data framework are urgently needed. Algorithmic Bias and Explainability: The “black box” nature of complex deep learning models presents a critical challenge in regulated environments. Regulatory bodies and clinicians require assurance that an AI-driven prediction is based on sound, non-biased evidence. Algorithmic bias, often stemming from non-diverse training data, could lead to therapies that are less effective or riskier for certain demographic groups. Thus, to manage the inherent risks of generative AI, the importance of traceability, transparency, and post-market performance monitoring should be emphasized.
- Patient Data Safety and Ethical Use: AI systems in CGT depend on large, high-dimensional patient datasets, often including genomic, immunological, and clinical information, which raises heightened concerns around privacy, consent, data provenance, and secondary use. Ensuring that these sensitive datasets are protected from misuse, securely integrated across platforms, and governed by transparent policies is essential to maintaining patient trust and meeting evolving regulatory expectations.
- Regulatory Uncertainty: Despite evolving position papers and guidelines from various regulatory bodies, there is a lack of clear, standardized regulatory frameworks to streamline the approval of Software as a Medical Device used in manufacturing or clinical decision support. For example, lack of harmonized AI terminology (i.e., no clear definition of explainability, understandability, and interpretability specific to the medical product area). It should be noted that most National Regulatory Authorities (NRAs) adopt a “horizontal AI definition”, where the highest risk management should be adopted. This may not be aligned with the sponsors/company’s approach. Without a clear definition, regulatory requirements could be ambiguous, and this makes risk management difficult to standardize.

Operational and Skill-based Hurdles: Implementing an intelligent manufacturing system requires significant investment in new digital infrastructure and, crucially, a new workforce with a blend of biomanufacturing expertise and data science/AI skills and necessary upskilling at the NRAs. However, it is important to note that some of the potential Regulatory and privacy constraints further complicate data use. The successful application of AI in CGT depends on the quality, interoperability, and governance of underlying data. AI models frequently integrate heterogeneous sources. including electronic health records (EHRs), genomic datasets, manufacturing records, and long-term follow-up data, yet these datasets are often fragmented, inconsistently structured, and subject to missingness or institutional bias. Such variability can limit model generalizability and compromise reproducibility across clinical sites [52].

Compliance with frameworks such as HIPAA in the United States and GDPR in Europe requires careful handling of protected health information, de-identification, and data-sharing practices [53]. In parallel, regulators increasingly expect transparency regarding data provenance, preprocessing, bias mitigation, and version control for AI systems used in drug development or manufacturing. Robust data governance and traceability are therefore foundational to ensuring credibility, fairness, and regulatory acceptance of AI-enabled CGT applications [54].

### 7.2. Lack of Benchmarking AI Against Established Methods in CGT Development

While artificial intelligence has demonstrated promise across vector design, translational modeling, and manufacturing optimization in CGT, direct benchmarking against established experimental and statistical methodologies remains limited. In many cases, AI-driven approaches are evaluated using retrospective datasets or simulated environments, with performance metrics such as predictive accuracy, area under the curve (AUC), or mean squared error reported in isolation. However, systematic comparisons against traditional mechanistic modeling, rule-based optimization, or classical design-of-experiments (DoE) frameworks are less frequently conducted.

As discussed in previous sections, in vector engineering, for example, machine learning–guided AAV capsid design has been shown to improve screening efficiency and identify functional variants beyond those discovered through rational mutagenesis alone. Yet head-to-head comparisons with directed evolution strategies across identical experimental conditions remain sparse. Similarly, in CAR-T construct optimization, AI-based sequence modeling and protein structure prediction (e.g., transformer architectures or deep graph neural networks) often outperform heuristic design approaches in silico, but prospective validation studies quantifying improvement in transduction efficiency, persistence, or clinical response are still emerging.

In manufacturing, reinforcement learning and digital twin–enabled control systems have demonstrated improved process stability and predictive quality monitoring compared with traditional statistical process control (SPC). However, robust benchmarking against established process analytical technology (PAT) frameworks under Good Manufacturing Practice (GMP) conditions is not yet widely reported. Classical DoE remains the regulatory gold standard for process characterization, and AI models are often used as complementary rather than replacement tools.

A key limitation is that many AI systems are evaluated on surrogate endpoints (e.g., transduction efficiency, capsid yield, phenotypic markers) rather than long-term clinical outcomes. Without prospective, multi-center validation, it remains difficult to quantify the incremental benefit of AI relative to conventional methods in terms of cost reduction, time-to-market acceleration, or product consistency. Although dedicated clinical trials explicitly designed to evaluate AI-enabled CGT development tools are still emerging, there is growing evidence that machine learning models can enhance decision making in toxicity prediction, resource scheduling, trial design and its regulations [46].

To advance the field, future research should incorporate standardized benchmarking frameworks that compare AI-enabled pipelines with traditional methodologies using shared datasets and predefined performance metrics. Here, AI can integrate more accurate measures from a historical control group in clinical design or in a real-world setting. Such hybrid approaches, such as combining mechanistic models, DoE, and machine learning, may ultimately provide the most robust and regulatory-acceptable solutions. Importantly, transparent validation protocols and reproducible evaluation criteria will be essential to justify broader regulatory and industrial adoption.

### 7.3. Opportunities and Future Outlook

Despite these challenges, the future of AI in CGT points towards a highly accelerated, personalized, and efficient ecosystem. In addition to the logistical challenges, the cost of CGT remains high and some of the AI-enabled approaches can help alleviate these challenges [55].

Accelerated Development and De-Risking: AI and digital twins can continue to shrink development timelines by enabling near-perfect in silico experimentation and optimization, leading to faster movement of novel therapies from the lab to patient approval. Enhanced Personalization: Technology may unlock the full potential of personalized medicine. AI-driven digital twins will move beyond generic models to enable real-time, patient-specific manufacturing and treatment strategies, tailoring every aspect of therapy to each patient’s biology.

- The Integration of Synthetic Biology: The convergence of AI and synthetic biology will allow for the rapid design and testing of highly sophisticated synthetic gene circuits. These circuits will enable programmatic control of cell fate, proliferation, and function in vivo, making stem cell and immune cell therapies more robust, reproducible, and clinically applicable [22].
- Collaboration: The translation of artificial intelligence into cell and gene therapy (CGT) development increasingly depends on strategic partnerships across academia, industry, technology providers, and regulatory stakeholders. Academic–industry collaborations have enabled machine learning–guided vector engineering and construct optimization, while biopharmaceutical–technology partnerships are advancing digital twin platforms, predictive quality analytics, and AI-driven manufacturing scheduling to improve operational efficiency and reduce batch failure risk [56] (REF). At the clinical interface, multicenter data-sharing initiatives are facilitating AI-based toxicity prediction and real-world evidence generation. These collaborative models not only accelerate technical innovation but also promote data standardization, cross-site validation, and regulatory alignment, all critical elements for scaling AI-enabled CGT from experimental implementation to sustainable, industry-wide practice.

### 7.4. Next Steps for the Industry

To realize this vision, the industry must prioritize three key areas:

- Build a Data-First Culture: Prioritize the collection of high-quality, structured, and richly annotated data from every step of the CGT lifecycle, from donor characteristics to long-term follow-up. Establishing industry-wide data standards is paramount.
- Embrace Strategic Partnerships and Collaborative Data Sharing: Given the scarcity of data, collaborations between technology vendors, academic research groups, and pharmaceutical companies are essential to pool resources, share insights, and build industry-wide benchmarks and standards for AI model performance.
- Start Small and Scale: Implement pilot projects to demonstrate the value of AI and digital twins on a small, focused scale before attempting a full-scale, facility-wide rollout.
- Engage with the NRAs early in the development process: Engaging early with regulatory authorities is essential in the development of CGTs due to the complexity, novelty, and evolving nature of these modalities. Early dialog enables sponsors to clarify regulatory expectations and align appropriate strategies for manufacturing, clinical development, and product characterization, particularly as many CGTs deviate from conventional product models. Proactive interaction helps address key challenges such as potency assay development, long-term safety follow-up, and vector or cell sourcing considerations, which, if left unresolved, can delay approvals. As regulatory frameworks are still maturing globally, establishing a shared understanding early on can significantly de-risk the development process.

## 8. Conclusions

The intersection of artificial intelligence and cell/gene therapy is contributing to medical breakthroughs. AI’s capacity to analyze complex biological data and optimize processes is a perfect complement to the innovative but intricate domain of CGT. As detailed above, AI is already shortening discovery timelines, personalizing treatment design, enhancing manufacturing reliability, and bolstering safety monitoring. These improvements address many of the pain points that have limited CGT’s scalability and accessibility. However, realizing AI’s full promise in this field requires careful navigation of challenges. Data must be harnessed effectively and responsibly; algorithms must undergo rigorous validation to meet safety standards; and scientists, engineers, and regulators must collaborate closely to keep progress aligned with patient welfare. The regulatory environment is catching up, with forward-looking frameworks like AI2ET pointing toward a more harmonized future regulating the AI-enabled ecosystem and not just the product. In conclusion, AI is not a panacea but rather a powerful enabling tool, one that, when integrated thoughtfully, can help CGT realize its revolutionary potential by delivering cures faster, more safely, and to more patients in need at an affordable cost. The coming years will be crucial as pilot successes evolve into industry norms, ultimately establishing AI-driven CGT as a new paradigm in biomedicine.

## Figures and Tables

**Table 1:** Key differences between Cell and Gene Therapy (CGT), Biologics, and Traditional Small Molecule Drugs across the discovery, development, manufacturing, and their lifecycle.

Attribute	Cell Therapies	Gene Therapies	Biologics	Small Molecules
Product Type	Living cells (autologous or allogeneic), minimally or more than minimally manipulated (e.g., MSCs, NK cells)	Genetic material delivered via viral/non-viral vectors; may involve in vivo or ex vivo modification	Large, complex proteins produced from living systems (e.g., MAb)	Small, chemically synthesized compounds
Molecular Size & Complexity	Extremely large; heterogeneous and variable		Large; structurally complex some cases well-characterized (e.g., rDNA protein products)	Small (~1 kDa); well-defined and reproducible
Production Approach	Personalized; scale-out manufacturing	Personalized (especially ex vivo); vector design critical	Standardized; Large scale production via bioreactors	Bulk synthesis; highly standardized
Manufacturing Process	Most complex; long cycles; chain-of-identity; cryopreservation	Complex; vector production + cell manipulation (ex vivo) or direct delivery (in vivo)	Complex; Fed-batch or continuous process	Simple; reproducible chemical synthesis and compounding
Stability, Supply Chain & Logistics	Most complex; patient-specific chain-of-identity; just-in-time delivery; least stable; requires cryotemps storage and supply	Complex; requires vector stability and cell viability (ex vivo) or formulation stability (in vivo)	Cold chain supply, relatively shorter shelf-life	Simplest; longer shelf-life, broad distribution; room temp storage
Follow-on Products	No generics/biosimilars; each product is unique		Biosimilars via comparability studies	Generics approved via BA/BE studies
Regulatory Pathway	Evolving but not many countries have regulatory framework		Well-established	Well-established
Cost of Production	Very high; customized, labor- and resource-intensive		High; living cell production from bioreactors, closed and aseptic process required	Lower; scalable, cost-effective manufacturing

**Table 2:** Translational Differences Between Cell Therapy & Gene Therapy.

Feature	Cell Therapy	Gene Therapy (Ex Vivo/In Vivo)
Source	Autologous or allogeneic cells	Patient or donor cells, or direct in vivo administration
Modification	Minimally or more than minimally manipulated (e.g., MSCs, iPSCs)	Gene editing or vector insertion
Potency Determinants	Viability, phenotype, functionality	Vector transduction (infectivity), transgene expression, delivery efficiency
Key Assay Focus	Identity, purity, immunomodulation	Vector copy number, product purity, off-target effects, durability
Regulatory Considerations	Focus on cell expansion & safety	Vector design, insertional mutagenesis, and immune response

**Table 3:** A Comparison of Standard and AI-Driven Approaches in the CGT Lifecycle.

Stage	Standard CGT Approach	AI-Driven CGT Approach
R&D/Design	Trial-and-error design of vectors; large experimental libraries, long cycles to optimize potency and safety.	AI predicts vector tropism, immune profile, and manufacturability; narrows candidates quickly; generative tools for novel capsids/CARs.
Preclinical & Translational	Animal models and small-scale assays; limited predictability for human outcomes.	Digital twins and in silico models simulate efficacy/toxicity; integration of omics and imaging for higher translational fidelity.
Manufacturing	Manual monitoring of bioreactors; batch failures detected late; corrective interventions after deviations.	Smart manufacturing with digital twins; real-time drift detection; predictive maintenance; adaptive control loops.
Clinical Trials	Broad eligibility, smaller populations, recruitment delays; adaptive designs are rare.	AI-enriched patient selection, adverse event risk prediction, adaptive trial designs; use of virtual/external controls.
Pharmacovigilance	Manual case intake, narrative writing, traditional signal detection in safety databases.	NLP-driven case processing; ML signal detection across multiple data streams (EHRs, registries, manufacturing data).
Regulatory Framework	Review based on static data packages; long cycles for post-approval monitoring.	Context-of-use validation of AI tools; real-time performance monitoring; adaptive regulatory engagement.

## Data Availability

The original contributions presented in this study are included in the article. Further inquiries can be directed to the corresponding author.
